# Low-temperature remote plasma enhanced atomic layer deposition of ZrO_2_/zircone nanolaminate film for efficient encapsulation of flexible organic light-emitting diodes

**DOI:** 10.1038/srep40061

**Published:** 2017-01-06

**Authors:** Zheng Chen, Haoran Wang, Xiao Wang, Ping Chen, Yunfei Liu, Hongyu Zhao, Yi Zhao, Yu Duan

**Affiliations:** 1 State Key Laboratory on Integrated Optoelectronics, College of Electronic Science and Engineering, Jilin University, Jilin 130012, China; 2 Computer Science and Technology Department, Jilin University, Changchun 130012, Jilin, China; 3 College of Science, Changchun University of Science and Technology, Changchun, 130012, China

## Abstract

Encapsulation is essential to protect the air-sensitive components of organic light-emitting diodes (OLEDs) such as active layers and cathode electrodes. In this study, hybrid zirconium inorganic/organic nanolaminates were fabricated using remote plasma enhanced atomic layer deposition (PEALD) and molecular layer deposition at a low temperature. The nanolaminate serves as a thin-film encapsulation layer for OLEDs. The reaction mechanism of PEALD process was investigated using an *in-situ* quartz crystal microbalance (QCM) and *in-situ* quadrupole mass spectrometer (QMS). The bonds present in the films were determined by Fourier transform infrared spectroscopy. The primary reaction byproducts in PEALD, such as CO, CO_2_, NO, H_2_O, as well as the related fragments during the O_2_ plasma process were characterized using the QMS, indicating a combustion-like reaction process. The self-limiting nature and growth mechanisms of the ZrO_2_ during the complex surface chemical reaction of the ligand and O_2_ plasma were monitored using the QCM. The remote PEALD ZrO_2_/zircone nanolaminate structure prolonged the transmission path of water vapor and smooth surface morphology. Consequently, the water barrier properties were significantly improved (reaching 3.078 × 10^−5^ g/m^2^/day). This study also shows that flexible OLEDs can be successfully encapsulated to achieve a significantly longer lifetime.

The wearable device market is rapidly expanding because of the development of cloud computing, smart sensors, and similar technologies, especially, flexible displays, with great advantages of lower production cost and higher durability, providing a combination of fashion and function^1^,^2^,^3^,^4^,^5^. Organic light-emitting diodes (OLEDs) are considered as the most promising candidate to produce displays because of the high flexibility of organic materials^6^,^7^. However, there are also some significant disadvantages including the problems of long-term stability and reliability of OLEDs because both the organic materials and cathode metals are vulnerable to atmospheric moisture and oxygen^8^,^9^. To take the full advantage of OLED potential, effective encapsulation is required to solve long-term stability and lifetime problems. More specifically, the water vapor transmission rate (WVTR) should be below 1 × 10^−6^ g/m^2^/day for successful commercial applications. The best developed technology is thin-film encapsulation (TFE), which has been recognized as a key component to achieve the commercial use of flexible OLEDs^10^. Using atomic layer deposition (ALD), TFE can provide very high-density films of inorganic materials such as SiNx, SiOx, AlOx, ZrOx, and TiOx)^11^,^12^,^13^,^14^,^15^,^16^,^17^. However, the deposition of thermally and mechanically sensitive materials requires a compatible low-temperature and low-radiation ALD^18^.

In previous study, we demonstrated that protective amorphous ZrO_2_ thin films can be produced at a low growth temperature by thermal ALD using O_3_ as the oxidant. Amorphous ZrO_2_ film avoided gas permeation through crystallite grain boundaries^19^. For the optimal low-temperature growth, the desired properties of ZrO_2_ based on TFE, such as a high gas barrier and flexibility, should be further improved.

The emerging molecular layer deposition (MLD) method can also be used for organic materials to fabricate high-quality thin films. This technique provides new types of highly uniform and conformal hybrid inorganic/organic thin films. The method involves the alternating exposure of inorganic and organic reactants on the substrate surface^20^,^21^. Both the MLD and ALD methods involve sequential and self-limiting surface reactions. They can also be used to fabricate highly conformal, continuous films without pinholes. Furthermore, the composite films consist of alternating layers of different materials with individual layers of nanometer-scale thickness. The latter is known as “nanolaminates”^22^. Hybrid nanolaminates have special properties. For example, they can extend the water vapor path and increase flexibility^23^,^24^. Therefore, an organic film produced by the MLD method has the potential to become a complementary method to improve the gas barrier of an inorganic film using an ALD system^25^,^26^.

In this study, a new family of hybrid inorganic/organic films known as “ZrO_2_/zircone” was investigated. The ZrO_2_ ALD and zircone MLD alloy TFEs were fabricated using the remote plasma enhanced ALD (PEALD) method at a low deposition temperature (80 °C). The PEALD is expected to increase the reactivity of the precursors, reduce the impurities, broaden the process window, and produce dense films^27^. Furthermore, the remote PEALD method has the advantage of a lower deposition temperature because the plasma enhances the reactivity of the precursors and a remote plasma reactor minimizes the effect of plasma-induced damage to the organic electronic device^28^. In the film growth process, the possible sequential reaction mechanisms of tetrakis(dimethylamino)zirconium (TDMAZ) and the O_2_ plasma were explored for the first time using an *in-situ* quartz crystal microbalance (QCM), an *in-situ* quadrupole mass spectrometer (QMS), and a Fourier transform infrared spssectrometer (FTIR). The atomic force microscopy (AFM) and X-ray diffraction (XRD) analyses showed that the hybrid ZrO_2_/zircone nanolaminate structure has a fine amorphous microscopic bulk and an almost homogeneous microscopic surface. Moreover, we found an interesting phenomenon: The film roughness shifted after modifying the relative number of ZrO_2_ ALD and zircone MLD cycles in the pulse sequence. In this approach, an extremely low WVTR of 3.078 × 10^−5^ g/m^2^ day was achieved using a 60 nm-ZrO_2_/zircone hybrid thin film (4 nm-ZrO_2_/1 nm-zircone) on a polyethylene glycol terephthalate (PET) substrate. The optimized hybrid film TFE of the structure exhibits a superior continuous operation lifetime of approximately 3.1-fold and 9.5-fold than that of the device TFE with pure ZrO_2_ and zircone-based TFE, respectively, under the controlled environment of 20 °C and a relative humidity of 60%.

## Results and Discussion

**Figure 1:**
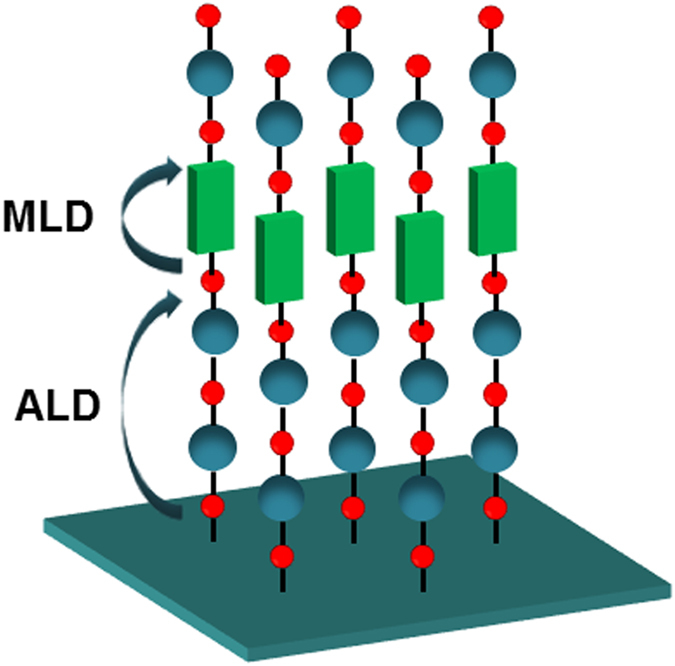
Structure of a hybrid inorganic/organic thin film fabricated using ALD/MLD technique.

ALD/MLD processes generally involve alternating metallic precursor and reactant exposure reactions that repeat the ALD and MLD processes^29^,^30^. This leads to a layer-by-layer growth as shown in Fig. 1. The ALD/MLD cycle can be separated into the following four steps including precursor pulsing and intermediate purging steps. Step 1: The first precursor is pulsed to the reactor, and it reacts with the surface species. Step 2: The excess precursor and possible byproducts are removed from the reactor by purging with an inert gas such as argon. Step 3: The second precursor is pulsed to the reactor, and it reacts with the surface species. Step 4: The excess precursor/possible byproducts are removed from the reactor.

**Figure 2:**
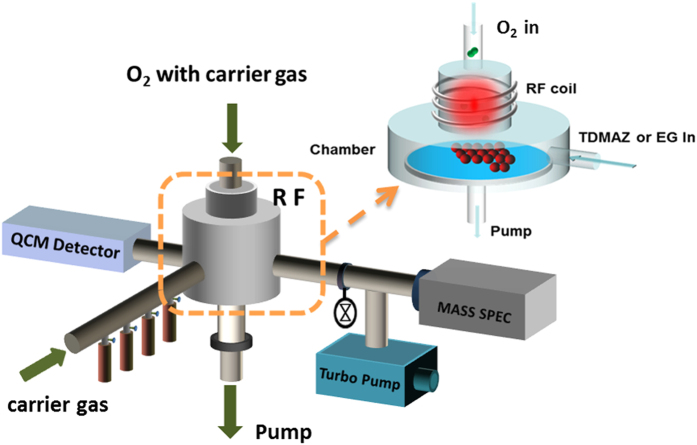
Schematic of the inlet and outlet connections of PEALD reactor.

Ideally, a monolayer thin film material is formed. To deposit thicker films, this basic ALD/MLD cycle is repeated as many times as needed to achieve the target film thickness. The remote PEALD method was used to fabricate 60-nm-thick zircone, ZrO_2_, and ZrO_2_/zircone nanolaminates at 80 °C. Moreover, ZrO_2_ films were prepared at deposition temperatures between 80–250 °C. Figure 2 shows a schematic diagram of a remote PEALD reactor. The metallic precursors entered through the side of the cavity via Ar (20 sccm). The O_2_ plasma was generated by a remote inductively coupled plasma generator at 100 W, and a constant O_2_ flow of 15 sccm was provided. O_2_ plasma with Ar (40 sccm) was applied from the top of the cavity to enter the chamber. The remaining unreacted precursor and byproducts were removed from the bottom of the mechanical pump. Although this setup provides a faster growth rate with O_2_ plasma, the fabricated thin films exhibited excellent conformity and uniformity, and they can effectively avoid plasma damage to organic electronic devices. The physical properties of TDMAZ and ethylene glycol (EG) were also analyzed. Because of their higher boiling point, the deposition at a lower temperature allows the provision of an appropriate amount of precursor. It also enables sufficient purging time to ensure that the precursor will not appear in the reaction cavity caused by precursor condensation phenomenon. Chemical vapor deposition (CVD) reaction can lead to the formation of pinholes, which greatly affected the properties of the encapsulation film.

Figure S1 shows the signals obtained in the QMS analysis and the change in molecular mass. Yoann Tomczak *et al*. studied lithium hexadimethyldisilazide and ozone ALD of lithium silicate^31^. A similar case showing the molecular or stoichiometric adsorption of LiHDMS followed its complete combustion. In this study, during the O_2_ plasma pulse, byproducts were observed at m/z = 44, 43, 30, 28, and 18, corresponding to CO_2_, CH_3_–N = CH_2_, NO, CO, and H_2_O, respectively. They are typical combustion byproducts of the O_2_ plasma process. Clearly, the signals originating from the fragments of the ligand (m/z = 15) indicate the presence of CH_3_ group in the plasma process. Therefore, the plasma process can be expressed by the following equations:


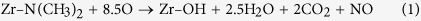



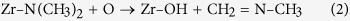


**Figure 3:**
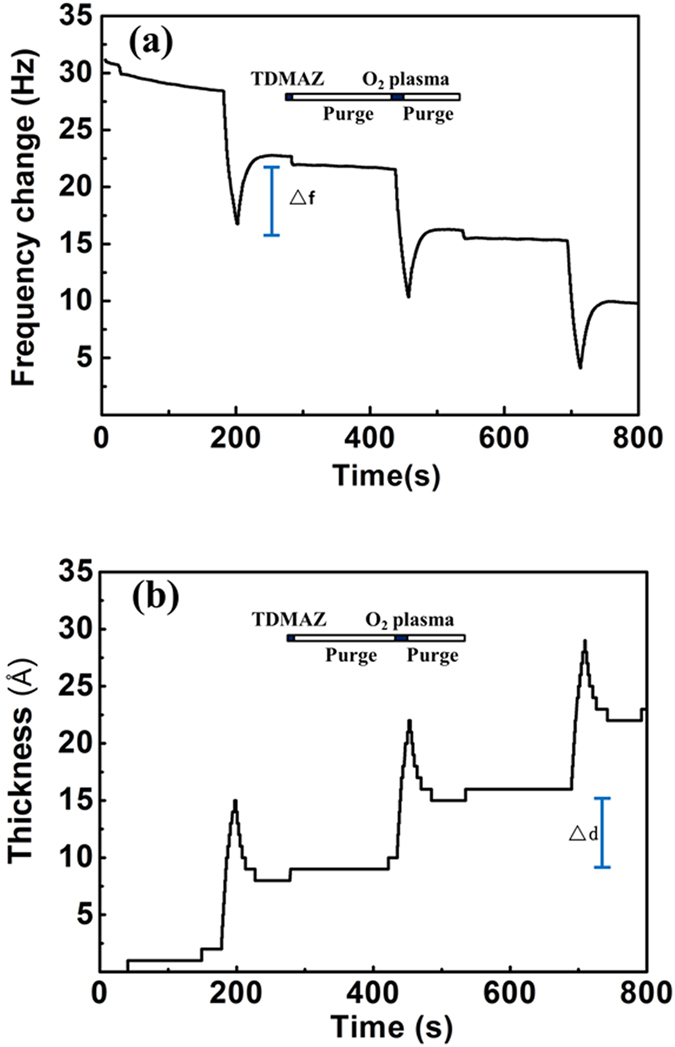
QCM results for three PEALD cycles of TDMAZ-O_2_ plasma process at 80 °C.

Figure 3 shows the QCM frequency change and thickness change for three full cycles of the deposition process at 80 °C. The crystal frequency changes with increasing ZrO_2_ film thickness can be expressed as follows:


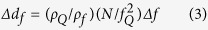


**Figure 4:**
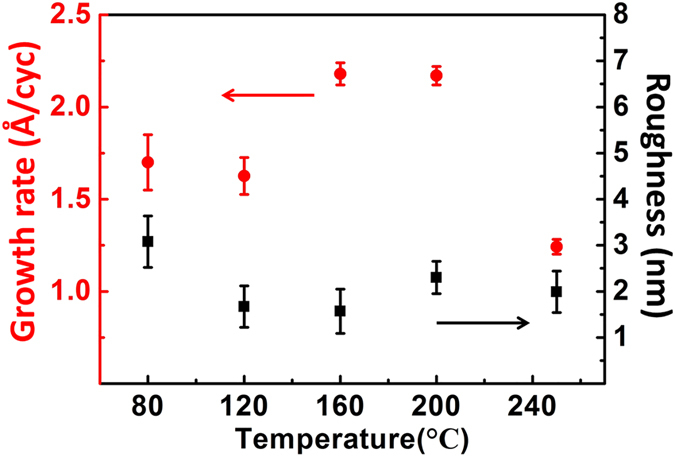
Growth rate and surface roughness of ZrO_2_ films as a function of substrate temperature.

Here, *ρ*_*Q*_ and *ρ*_*f*_ are the quartz density (2.65 g/cm^3^) and deposited film density (the density of ZrO_2_ is 5.6 g/cm^3^), respectively, *N* is a frequency constant (1670 KHz·mm), and *f*_*Q*_ is the quartz crystal intrinsic vibration frequency. The observed film thickness (△*d*) and the change in frequency (△*f*) indicate a linear relationship. Because the resolution ratio of the equipment is 1 Å, the density of ZrO_2_ was reduced by a factor of 1 over 4 of the original density. Thus, the change in film thickness increased four times. The crystal frequency and thickness curves are highly consistent, indicating the uniform increment of thickness in each cycle. Figure 4 shows the dependence of the growth rate and roughness of ZrO_2_ film as a function of deposition temperature, which varied between 80–250 °C at a deposition pressure of 0.15 Torr. The nominal thickness of ZrO_2_ was thoroughly controlled at the atomic scale during the processing, using the values 1.714, 1.5, 2.18, 2.17, and 1.24 Å/cyc at 80, 120, 160, 200, and 250 °C, respectively. The film uniformity of ZrO_2_ films was found to be more stable at higher deposition temperatures. At 80 °C deposition temperature, the roughness is clearly higher than that at other temperatures. In this experiment, the growth per cycle outside the temperature window was lower than that at the window temperature, because of precursor noncondensation resulting from the reasonable control of precursor flows and prolonged purging times. Growth rate is more stable between 160–200 °C, and at a higher growth rate, the process temperature ranges in the ALD window temperature. However, at 250 C, the growth rate decreased significantly due to the decomposition of precursor and desorption^29^,^32^,^33^.

**Figure 5:**
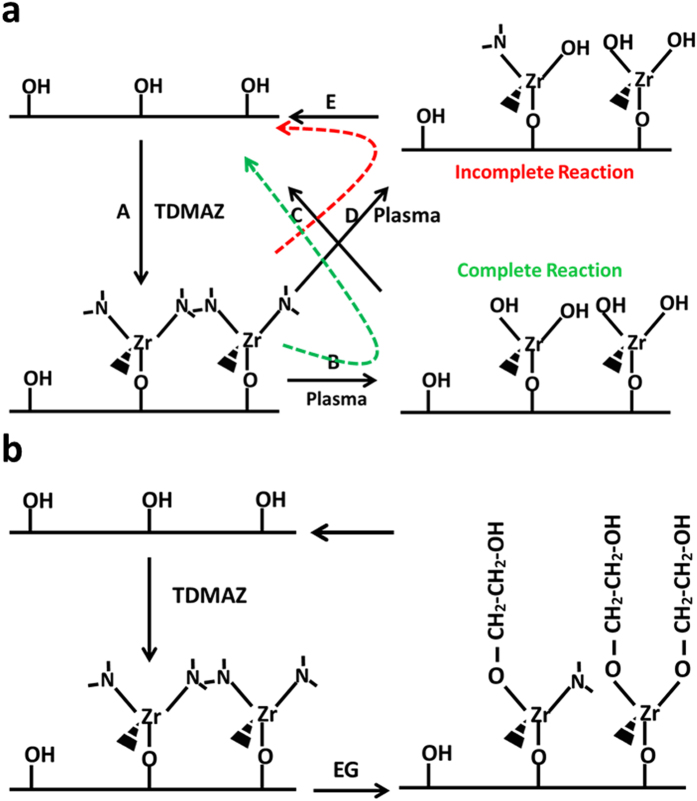
Possible several reaction (**a**) pathways for TDMAZ and O_2_ plasma, Green dashed line: complete reaction (path A---path B---path C); Red dashed line: incomplete reaction (path A---path D---path E); (**b**) pathway for TDMAZ and EG.

Figure 5(a) shows the possible adsorption and reaction steps of the TDMAZ precursor and O_2_ plasma on substrate surface. Ideally, for every hydroxyl group reaction with TDMAZ, each dimethylamino group will be attached to the hydroxyl groups in the O_2_ plasma reaction. However, this is not the case as expected; our experiments show that methyl and hydroxyl groups were present in the film.

**Figure 6:**
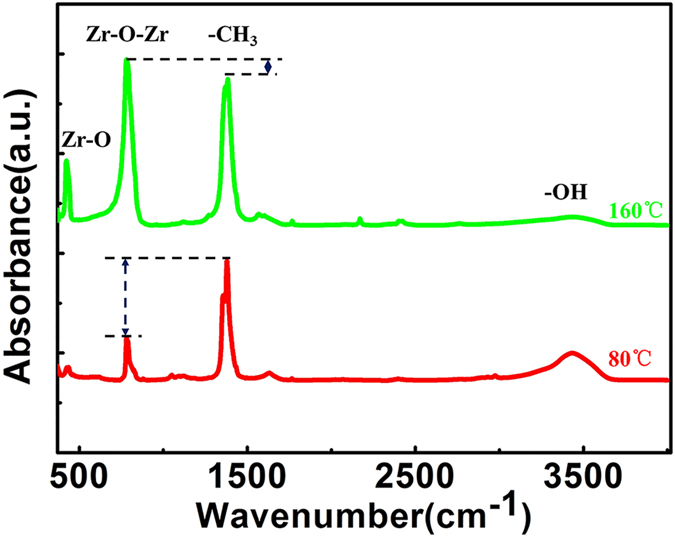
FTIR spectra of 100-cycle ZrO_2_ thin film deposited using TDMAZ and O_2_ plasma.

Path A shows that the TDMAZ precursor can react with the surface hydroxyl groups by exchanging dimethylamino groups, however, hydroxyl groups do not fully adsorb TDMAZ. Moreover, the green line direction shows that all the dimethylamino groups will be attached to the hydroxyl groups in the O_2_ plasma reaction under ideal conditions. Nevertheless, at a low temperature, a part of dimethylamino groups does not participate in the reaction (red line direction). This assumption was confirmed by detecting a large amount of dimethylamino by FTIR. Then, they are transferred to the TDMAZ and start the next cycle. Figure 5(b) shows the pathway for TDMAZ and EG. We assumed that double reactions exist, although no direct evidence was observed in this experiment. The FTIR spectra of the deposited ZrO_2_ film at 80 °C and 160 °C are shown in Fig. 6. The broad bands around 3400 cm^−1^ can be attributed to the stretching vibration of H_2_O. The peak at 435 cm^−1^ can be assigned to the Zr–O bond in ZrO_2_. The peak in the region 780–790 cm^−1^ can be attributed to asymmetric vibration of the Zr–O–Zr bond. The sharp peak at 1375–1380 cm^−1^ can be assigned to the remaining CH_3_, indicating an incomplete reaction of the Zr–N(CH_3_)_2_ moiety with O_2_ plasma. However, the peak density of Zr–O–Zr is stronger than that of CH_3_ at 160 °C. On the other hand, the peak of CH_3_ is stronger than the Zr–O–Zr peak. Clearly, the reaction occurs more readily with increasing temperature^34^.

**Figure 7:**
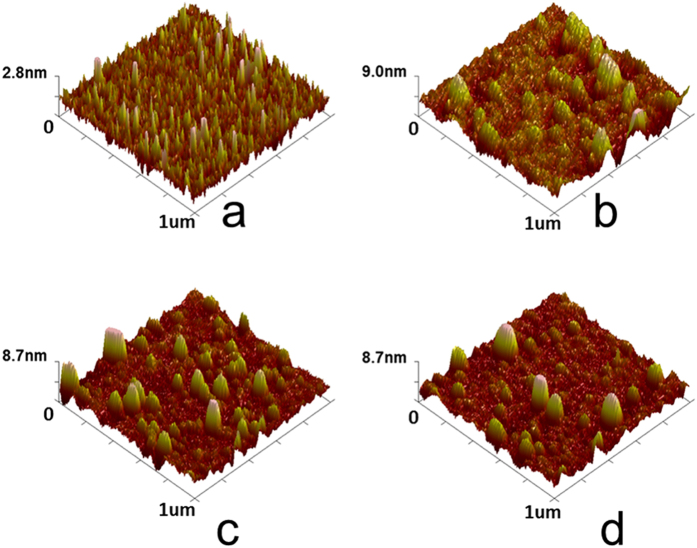
Atomic force microscopy images: (**a**) EG-based zircone (**b**) O_2_ plasma-based ZrO_2_, and (**c**) and (**d**) ZrO_2_/zircone nanolaminate (embedded 4-nm zircone and embedded 1-nm zircone, respectively) at 80 °C.

To investigate the surface morphology of the thin films, the 60 nm- zircone/ZrO_2_/hybrid nanolaminates (8 nm-ZrO_2_/2 nm-zircone and 4 nm-ZrO_2_/1 nm-zircone nanolaminates) were deposited on clean Si substrates at 80 °C. A scanned area of 1 × 1 μm^2^ was observed using an AFM (Fig. 7). Pure zircone and ZrO_2_ films were also characterized as the references. As shown in Fig. 7(a), the pure zircone film has superior smoothness and less rough surface (RMS = 0.43 ± 0.04 nm). The pure ZrO_2_ surface film was relatively rough with RMS = 3.18 ± 0.48 nm (Fig. 7(b)). The RMS variation of the ZrO_2_/zircone nanolaminates increased rapidly from 1.82 ± 0.16 nm for ZrO_2_/zircone nanolaminates (8:2) (Fig. 7(c)) to 0.93 ± 0.12 nm for ZrO_2_/zircone nanolaminates (4:1) (Fig. 7(d)). The increase was probably because the organic zircone acted as a buffer layer that potentially overcame the defects with a smooth interlayer for ZrO_2_ film growth^35^.

The permeation of water vapor is significantly affected by the crystallinity of thin films^26^,^36^. Therefore, the XRD spectra of 60-nm-thick zircone and ZrO_2_ together with ZrO_2_/zircone hybrid thin films at 80 °C were obtained. Figure S2 shows the XRD spectra of the permeation barrier layers deposited using the PEALD method. All the permeation barrier layers showed an amorphous structure without a clear crystallization peak, indicating that the 60-nm-thick ZrO_2_ films deposited using low-temperature PEALD have a lower surface crystallinity.

To evaluate the permeability of a ZrO_2_ film as the water diffusion barrier deposited using the PEALD method, the WVTR was determined using the electrical Ca corrosion test with a 100-nm-thick Al electrode and a 200-nm-thick Ca layer with an area of 1 × 1 cm^2^. In the test, the Ca layer was deposited on a glass substrate connected with two aluminum electrical leads. The Ca layer was then covered with a thin film barrier. The electrical measurements were performed using two electrodes connected with a source-measure-unit probe to an Agilent 2920 source meter. During the Ca corrosion test measurement, the electrical conductance through the Ca layer decreased as Ca was oxidized by the water vapor penetrating the barrier film. The slope d(1/R)/dt was used to calculate the effective WVTR values using the following equation:


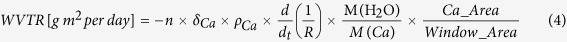


here, n is the molar equivalent of the degradation reaction, assumed as n = 2, based on the chemical reaction of Ca with water. *δ*_*ca*_ is the Ca resistivity (3.4 × 10^−8^ Ω m); *ρ*_*Ca*_ is the Ca density (1.55 g/cm^3^); M(H_2_O) and M(Ca) are the molar masses of water vapor and Ca, respectively. The ratio between the area of the Ca and the area of the window for water permeation is 1. (1/R) is the conductance of our samples during the tests. Figure S3 shows the results of the WVTR measurement for the permeation barrier layers produced in this experiment. The conductance change of the Ca devices was measured as a function of water exposure time in the atmospheric environment at 20 °C and 60% RH. The WVTR value of 60-nm zircone layer was 0.1033 g/m^2^/day, which is a very bad result for TFE. The 60-nm ZrO_2_ single layer shows a positive effect for the encapsulation, and the obtained WVTR value was 1.39 × 10^−4^ g/m^2^/day. According to our series of experiments, lower-temperature ZrO_2_ showed the presence of dimethylamino and hydroxyl groups because of the incomplete reaction. A lack of crystalline ZrO_2_ leads to the formation of a dense thin film. Zircone can prolong the water vapor penetration path and limit the appearance of holes. The WVTR values for the ZrO_2_/zircone nanolaminates (embedded 4-nm zircone and 1-nm zircone) were 7.619 × 10^−5^ g/m^2^ day and 3.078 × 10^−5^ g/m^2^ day, respectively. This result shows that the ZrO_2_/zircone nanolaminates (4:1) deposited at 80 °C has a better water barrier.

**Figure 8:**
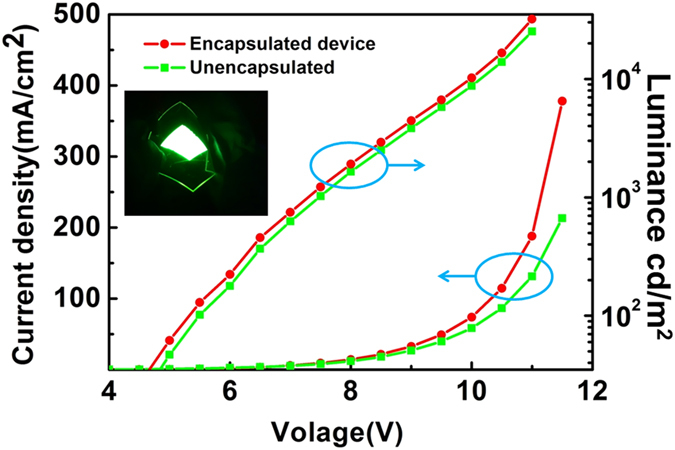
Luminance and current vs. operating voltage of bare and encapsulated OLEDs with ZrO_2_ thin film. The inset shows a bent device on a PET substrate.

To study the encapsulation properties and plasma effect on organic electronics, flexible OLEDs were fabricated using a PET substrate. The lifetime of the OLEDs was measured for an initial luminance of approximately L_0_ = 1000 cd/m^2^. The electrical and emission characteristics of the devices were measured simultaneously using an Agilent 2920 source meter and a Minolta luminance meter LS-110 in air at 20 °C and 60% RH. Figure S4 shows typical plots of normalized luminance vs. operating time of the OLEDs with different encapsulations. The dependence of luminance vs. operating time was measured with a constant DC voltage. The OLED with a hybrid TFE had a lifetime of 182 h with an elapsed time of instantaneous luminance decaying to 50% from its initial luminance. Then, a 60-nm TFE of ZrO_2_ nanolaminate was to fabricate a flexible OLED (Fig. 8). The inset of Fig. 8 shows a flexible OLED with TFE in the bending test at a radius of 5 mm. Compared to bare OLED, the TFE devices showed no effect in a remote plasma chamber using the PEALD method. It is a requisite for the encapsulation of organic electronic devices. The current density and luminance values obtained from the remote PEALD-processed devices were larger than that of the nonencapsulated devices at the same voltage. Moreover, only a slight difference in the L–V behavior before and after encapsulated devices was observed using the same OLED device sample. No increase in the number of small black spots was observed under a microscope. O_2_ plasma damage was very limited because the PEALD process involved a short plasma exposure time and large intervals between the O_2_ plasma steps. We assume that the small degree of change is closely associated with the thermal annealing during the ALD process.

## Conclusions

Zirconium is a key component in functional layers for device encapsulation and thermal barrier coatings because of its thermal, mechanical, optical, and electrical properties. In this study, amorphous and homogeneous ZrO_2_/zircone hybrid nanolaminate films were successfully synthesized for OLED encapsulation using the remote PEALD method at a low temperature (80 °C). The physicochemical studies using *in-situ* QCM, QMS, and FTIR indicate that a combustion-like reaction occurs during the remote O_2_ plasma process, allowing us to determine the dependence of the product and its morphology on temperature. The surface roughness of the hybrid nanolaminate films with a zircone ultrathin layer inserted into the ZrO_2_ thin film was 0.93 ± 0.12 nm at the same thickness. An increase in the ratio of the organic layer increased the surface roughness of the ZrO_2_/zircone hybrid nanolaminate films. Moreover, the WVTR of hybrid thin films, compared to 60-nm-thick ZrO_2_ decreased from 1.39 × 10^−4^ g/m^2^/day to 3.078 × 10^−5^ g/m^2^/day with a higher number of stacked layers, indicating that the ultrathin thin films of zircone blocked or prolonged the path of water vapor and oxygen through the bulk ZrO_2_. Because of very low permeation rates, low process temperature, and its mechanical compatibility, the nanolaminate provided improved protection for flexible organic electronic devices.

## Methods

The hybrid ZrO_2_/zircone nanolaminate was deposited using the remote PEALD method (PEALD-150A, Kemicro). All the substrates were thoroughly cleaned with acetone, alcohol, and deionized water successively. They were then placed in an ultrasonic bath, first in acetone and then in alcohol. The inorganic barrier layers were made of ZrO_2_ using TDMAZ and O_2_ plasma as the precursors. The organic barrier layers were fabricated using TDMAZ and EG as the precursors by the MLD method in the same reactor. TDMAZ and EG were kept at 85 and 95 °C, respectively. The pressure was 0.15 Torr, and high-purity Ar (60 sccm, 99.999%) was used as the carrier gas for the precursors, and high-purity O_2_ (15 sccm, 99.999%) was used as the O_2_ plasma source. A single ZrO_2_ cycle comprised the following steps: TDMAZ dose (0.02 s), Ar purging (150 s), O_2_ plasma treatment with 100 W RF plasma power (20 s), and Ar purging (60 s). The sequence of pulses for one-cycle deposition of zircone was as follows: TDMAZ dose (0.02 s), Ar purging (150 s), EG dose (0.02 s), and Ar purging (200 s). The thickness of the thin films deposited on the clean Si substrate as well as the frequency change was measured *in situ* using the QCM. The present gas species in the reactor during the PEALD process was investigated using the QMS. The pressure in the QMS chamber was about 1 × 10^−5^ Torr. The samples for FTIR analysis were grown with a 100-cycle ZrO_2_ deposition and scanned between 350 and 4000 cm^−1^ at a resolution of 4 cm^−1^. The root-mean-square (RMS) roughness and surface features of the films were observed by AFM analysis. The phase and crystallinity of the films were investigated using XRD analysis. The value of WVTR was measured using the Ca corrosion test method on glass substrate. We deposited the barrier film on the clean PET substrate, on which then we fabricated the OLEDs, finally to encapsulate the devices. Namely, both sides of the device consisted of encapsulation films. The prototypical structure of the OLEDs fabricated via vacuum thermal deposition can be described as follows: 100-nm-Al/2-nm-thick MoO_3_ layer/30-nm-thick 4,4′,4′′-tris (*N*-3(3-methylphenyl)-*N*-phenylamino) triphenylamine (m-MTDATA) as the hole injection layer/20-nm-thick N,N′-biphenyl-N,N′-bis(1-naphenyl)-[1,1′-biphenyl]-4,4′-diamine (NPB) as the hole transport layer/30-nm-thick tris-(8-hydroxyquinoline) aluminum (Alq3) with 2% C545T as the light-emitter/20-nm-thick Alq3 as the electron transport layer-/1-nm-thick Liq capping with a 15-nm-thick Ag cathode. The sample was then transferred into a nitrogen glove box for using the PEALD system. The electrical characteristics of the devices were measured using an Agilent 2920 source meter (Agilent Technologies, Inc., Santa Clara, CA, USA) at room temperature.

## Additional Information

**How to cite this article**: Chen, Z. *et al*. Low-temperature remote plasma enhanced atomic layer deposition of ZrO_2_/zircone nanolaminate film for efficient encapsulation of flexible organic light-emitting diodes. *Sci. Rep.* **7**, 40061; doi: 10.1038/srep40061 (2017).

**Publisher's note:** Springer Nature remains neutral with regard to jurisdictional claims in published maps and institutional affiliations.

## Supplementary Material

Supplementary Information
