# Supplementary material for: Low-temperature remote plasma enhanced atomic layer deposition of ZrO2/zircone nanolaminate film for efficient encapsulation of flexible organic light-emitting diodes
Source: Sci Rep. 2017 Jan 6;7:40061. doi: 10.1038/srep40061 (PMC5216332; doi:10.1038/srep40061)
Supplement: Supplementary Information [file srep40061-s1.doc]

**Low-temperature remote plasma enhanced atomic layer deposition of ZrO_2_/zircone nanolaminate film for efficient encapsulation of flexible organic light-emitting diodes**

**Zheng Chen^1^, Haoran Wang^1^, Xiao Wang^1^, Ping Chen^1^, Yunfei Liu^1,2^, Hongyu Zhao^1^, Yi Zhao^1^, Yu Duan^1,3^†**

1. State Key Laboratory on Integrated Optoelectronics, College of Electronic Science and Engineering, Jilin University, Jilin 130012, China
2. Computer Science and Technology Department, Jilin University, Changchun 130012, Jilin, China
3. College of Science, Changchun University of Science and Technology, Changchun, 130012, China


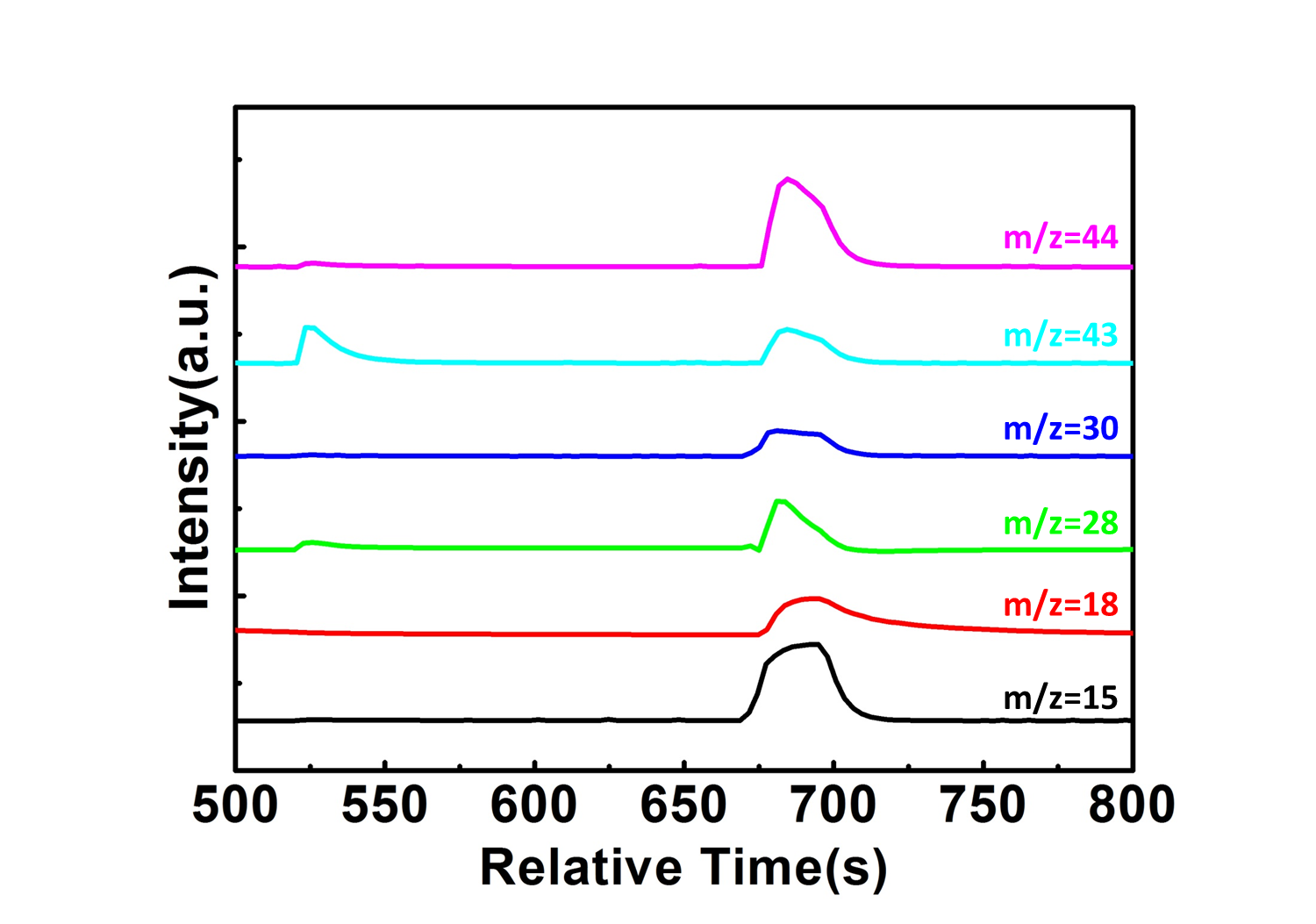


Figure S1: QMS signals for important by-products of the ZrO_2_ deposition process.


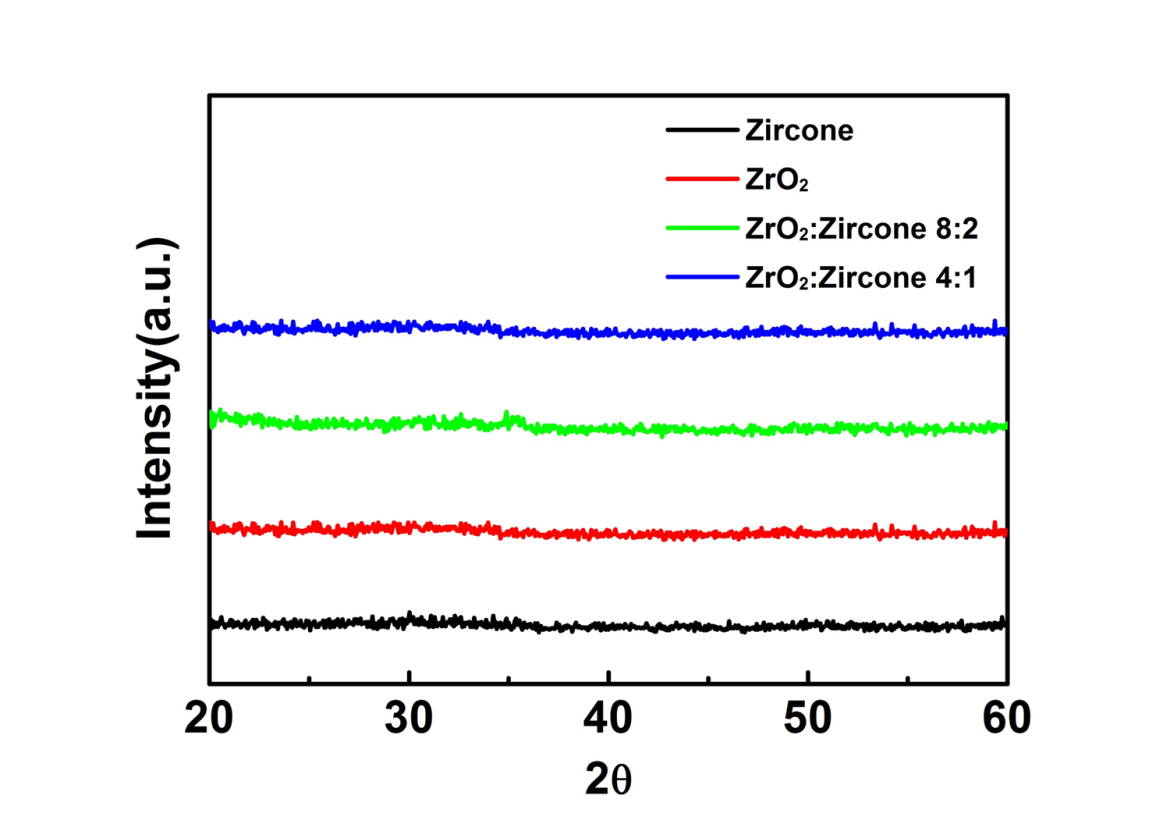


Figure S2: X-Ray diffraction patterns of ZrO_2_, Zircone and ZrO_2_/Zircone nanolaminate (embedded 4 nm Zircone and embedded 1 nm Zircone， respectively) with a total of 60nm at 80℃.


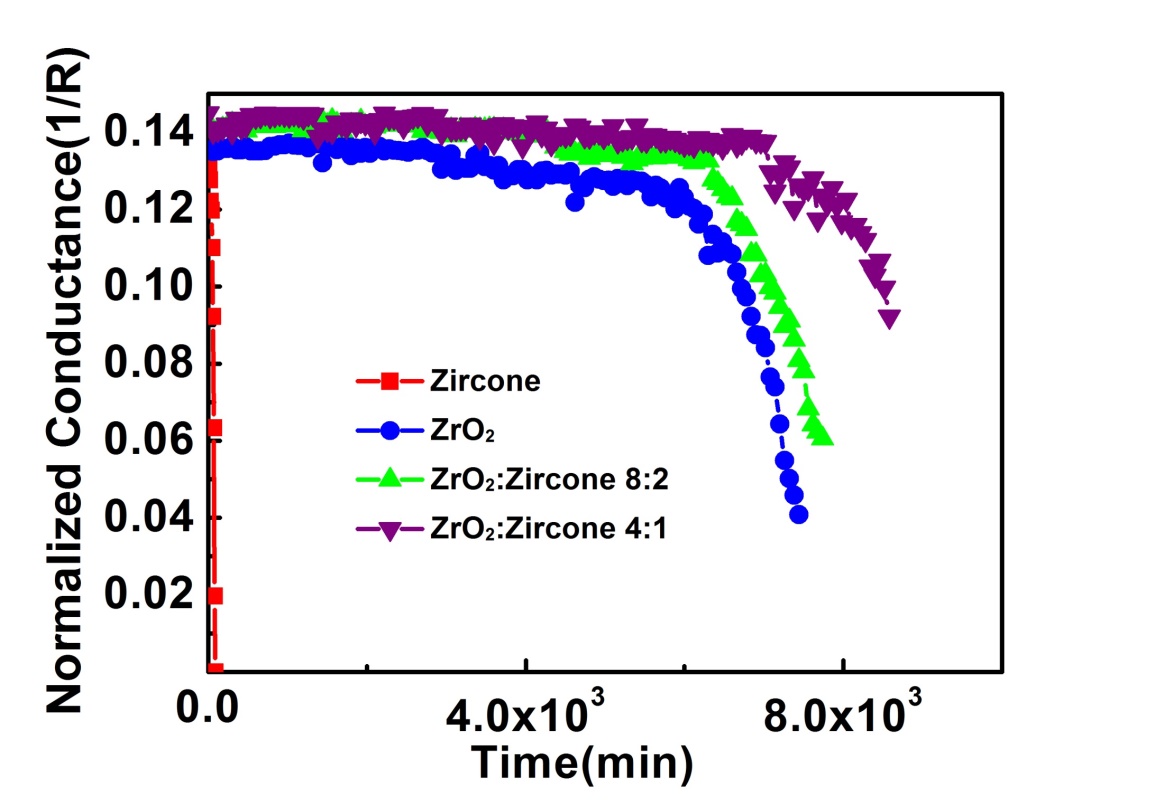


Figure S3: The conductance changes of Ca as a function of time for ZrO_2_ and the hybrid nanolaminate film were measured at 20℃, 60 % RH.


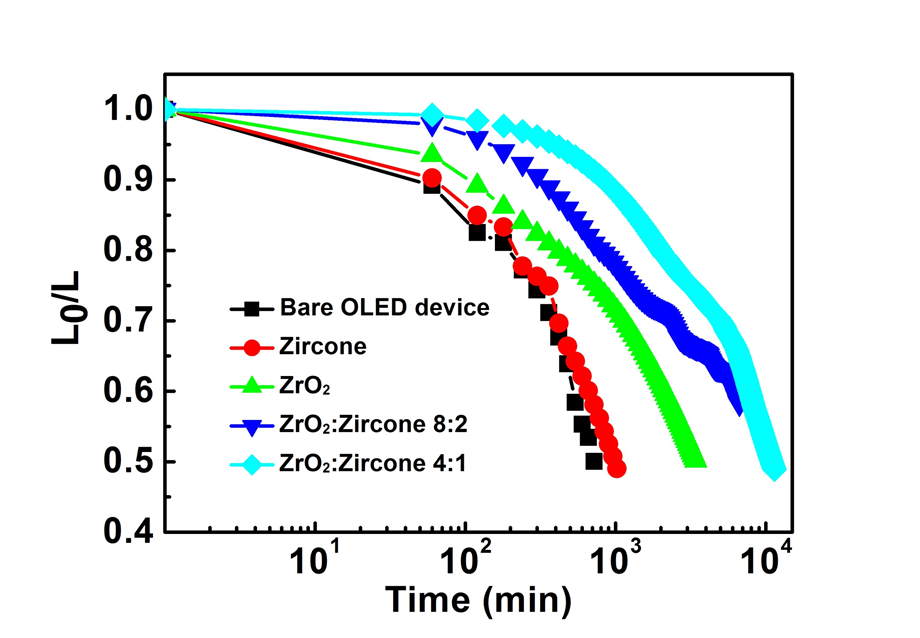


Figure S4: Normalized experimental luminance decay as a function of continuous operating time for OLEDs encapsulated with film at ambient conditions of 20 ℃, 60 %RH.
